# Modified Ferguson hemorrhoidectomy for grade II–IV hemorrhoids offers low recurrence and complication rates: a retrospective cohort study

**DOI:** 10.3389/fmed.2025.1710244

**Published:** 2026-01-12

**Authors:** Run-Yi Geng, Lu Yin, Yong-Qing Cao, Xiao Shen, Ya-Qing Ding, Yi-Bo Yao, Chen Wang

**Affiliations:** 1 Department of Anorectal Surgery, Longhua Hospital Shanghai University of Traditional Chinese Medicine, Shanghai, China; 2 Department of Anorectal, Shanghai Fourth People's Hospital, School of Medicine, Tongji University, Shanghai, China

**Keywords:** hemorrhoids, hemorrhoidectomy, modified Ferguson, recurrence, postoperative complications

## Abstract

**Purpose:**

This study aimed to evaluate the efficacy and safety of modified Ferguson hemorrhoidectomy (MFH) compared with conventional Milligan-Morgan hemorrhoidectomy (MMH) for grade II–IV hemorrhoids.

**Methods:**

A retrospective cohort of patients undergoing MFH or MMH between September 2020 and August 2021 was reviewed. The primary outcomes were healing time and recurrence after at least 24 months of follow-up. Secondary outcomes included Hemorrhoidal Disease Symptom Score (HDSS), postoperative complications, patient satisfaction, and quality-of-life assessment.

**Results:**

A total of 515 patients were enrolled (MFH, *n* = 254; MMH, *n* = 261) with a median follow-up of 29 months. The recurrence rate was significantly lower in the MFH group compared with MMH (1.2% vs. 5.0%, *p* = 0.013), with Kaplan–Meier analysis confirming superior recurrence-free survival (98.8% vs. 95.0% at 36 months). Healing time was shorter with MFH (26.3 ± 4.3 vs. 30.9 ± 3.5 days, *p* < 0.001). MFH reduced the risk of postoperative edema (2.4% vs. 9.6%, *p* = 0.01; OR 0.23, 95% CI 0.09–0.56) but showed a higher incidence of urinary retention (3.9% vs. 1.1%, *p* = 0.04). No anal stenosis or fecal incontinence was observed in either group. Patient satisfaction was higher in MFH (9.7 ± 0.7 vs. 9.4 ± 1.2, *p* = 0.002), and improvements in HDSS and SHSHD scores were sustained during long-term follow-up. Multivariate regression confirmed MFH as an independent protective factor against recurrence (adjusted OR 0.26, 95% CI 0.08–0.91, *p* = 0.035).

**Conclusion:**

Modified Ferguson hemorrhoidectomy is a safe and effective technique for grade II-IV hemorrhoids, offering lower recurrence, shorter healing, fewer complications, and higher satisfaction compared with Milligan-Morgan hemorrhoidectomy.

## Introduction

Hemorrhoidal disease (HD) is among the most prevalent anorectal conditions worldwide, with considerable negative impact on quality of life (1, 2). Surgical intervention is typically indicated when conservative treatments prove ineffective (3, 4). Hemorrhoidectomy remains the cornerstone surgical treatment, performed primarily via either the closed [Ferguson (5)] or open [Milligan–Morgan (6)] technique. The Ferguson approach is associated with superior primary healing, reduced postoperative bleeding, less scarring, and better preservation of anal sensation compared to the Milligan–Morgan procedure (7–10). Nonetheless, it is not without limitations, including risks of pain, wound infection, and suture line dehiscence (7, 11, 12). In response to these drawbacks, we developed a modified Ferguson hemorrhoidectomy (MFH), aimed at minimizing postoperative complications without compromising efficacy. Key modifications include the integration of superior hemorrhoidal artery ligation into the conventional Ferguson procedure.

The incorporation of superior hemorrhoidal artery ligation is derived from the pathophysiology of hemorrhoidal bleeding. A primary component of the disease process is the hypertrophy and increased blood flow of the hemorrhoidal arterial plexus. By ligating the terminal branches of the superior hemorrhoidal artery supplying the pathologic cushions prior to excision, we hypothesize a significant reduction in arterial inflow. This devascularization effect is intended to minimize the risk of postoperative bleeding from the pedicle, a potentially serious complication often resulting from the slippage of a ligature or eschar separation from a highly vascularized wound bed.

Earlier institutional experience indicated that MFH reduces both bleeding and acute thrombotic events. This retrospective study was therefore designed to compare the clinical outcomes and safety profile of MFH versus conventional Milligan–Morgan hemorrhoidectomy (MMH).

## Methods

### Ethical considerations

This retrospective cohort study was conducted in accordance with the Strengthening the Reporting of Observational Studies in Epidemiology (STROBE) guidelines (13). And it was approved by the Ethics Committee of Longhua Hospital (approval number: 2022LCSY102).

### Study population

We retrospectively reviewed patients who underwent either MFH or MMH at Longhua Hospital between September 1, 2020, and August 31, 2021. Eligible patients were contacted by telephone and invited to attend a follow-up proctological examination. They were also asked to complete questionnaires regarding symptom recurrence and satisfaction with surgical outcomes. Inclusion criteria comprised: age between 18 and 65 years, a diagnosis of grade II–IV internal hemorrhoids based on Goligher’s classification (14), who were indicated for surgical intervention due to the failure of conservative or office-based procedures (e.g., rubber band ligation, sclerotherapy) or due to the severity of symptoms (e.g., large prolapse, persistent bleeding), and a minimum follow-up period of two years. Patients with previous anorectal surgery: Patients with a history of prior hemorrhoid surgery were included in the analysis for several reasons. First, this reflects real-world clinical practice where a proportion of patients present with recurrent or persistent symptoms requiring reoperation. Second, their inclusion allows for the evaluation of surgical outcomes in a more challenging subgroup, thereby testing the robustness of both techniques. To address potential confounding, we performed subgroup analysis which specifically compared recurrence rates in patients with prior surgery, and multivariate regression was used to adjust for prior surgery as a covariate, confirming that surgical technique remained an independent predictor of outcomes. Exclusion criteria included concomitant anorectal pathologies (such as anal fissure, perianal fistula, or perianal abscess), diagnosed mental illness, and insufficient clinical or follow-up data.

Although total colonoscopy is not routinely mandated for all patients undergoing hemorrhoidectomy in our institution, patients with symptoms suggestive of colorectal malignancy (e.g., unexplained weight loss, change in bowel habit, family history of colorectal cancer, or iron-deficiency anemia) were referred for preoperative colonoscopy and excluded if malignancy was detected. All enrolled patients had symptoms attributable primarily to hemorrhoidal disease and no clinical indicators of underlying malignancy.

The choice of surgical procedure (MFH or MMH) was based on the operating surgeon’s preference and clinical judgment, as there was no institutional protocol mandating one technique over the other during the study period.

**Figure 1:**
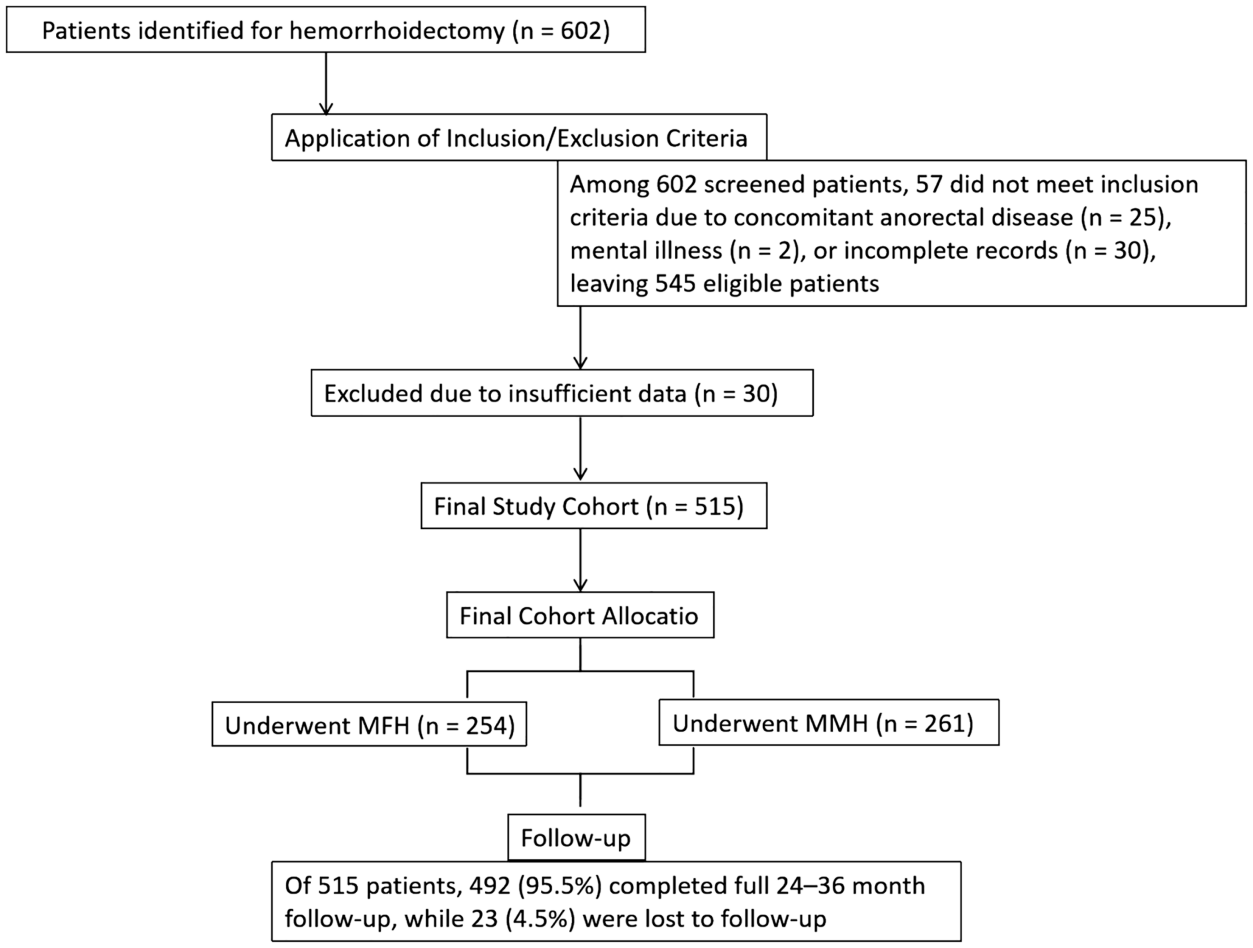
Patient selection flowchart.

A total of 602 patients were identified as having undergone hemorrhoidectomy (MFH or MMH) during the study period. After applying the inclusion and exclusion criteria, 545 patients were deemed eligible. Of these, 30 patients were excluded due to insufficient clinical or follow-up data, resulting in a final analyzed cohort of 515 patients (MFH, *n* = 254; MMH, *n* = 261). The participant flow is summarized in Figure 1.

### Perioperative management and surgical technique

Bowel preparation was achieved using glycerin enemas administered on the morning of surgery. All procedures were performed under spinal anesthesia, with patients placed in the lithotomy position.

**Figure 2:**
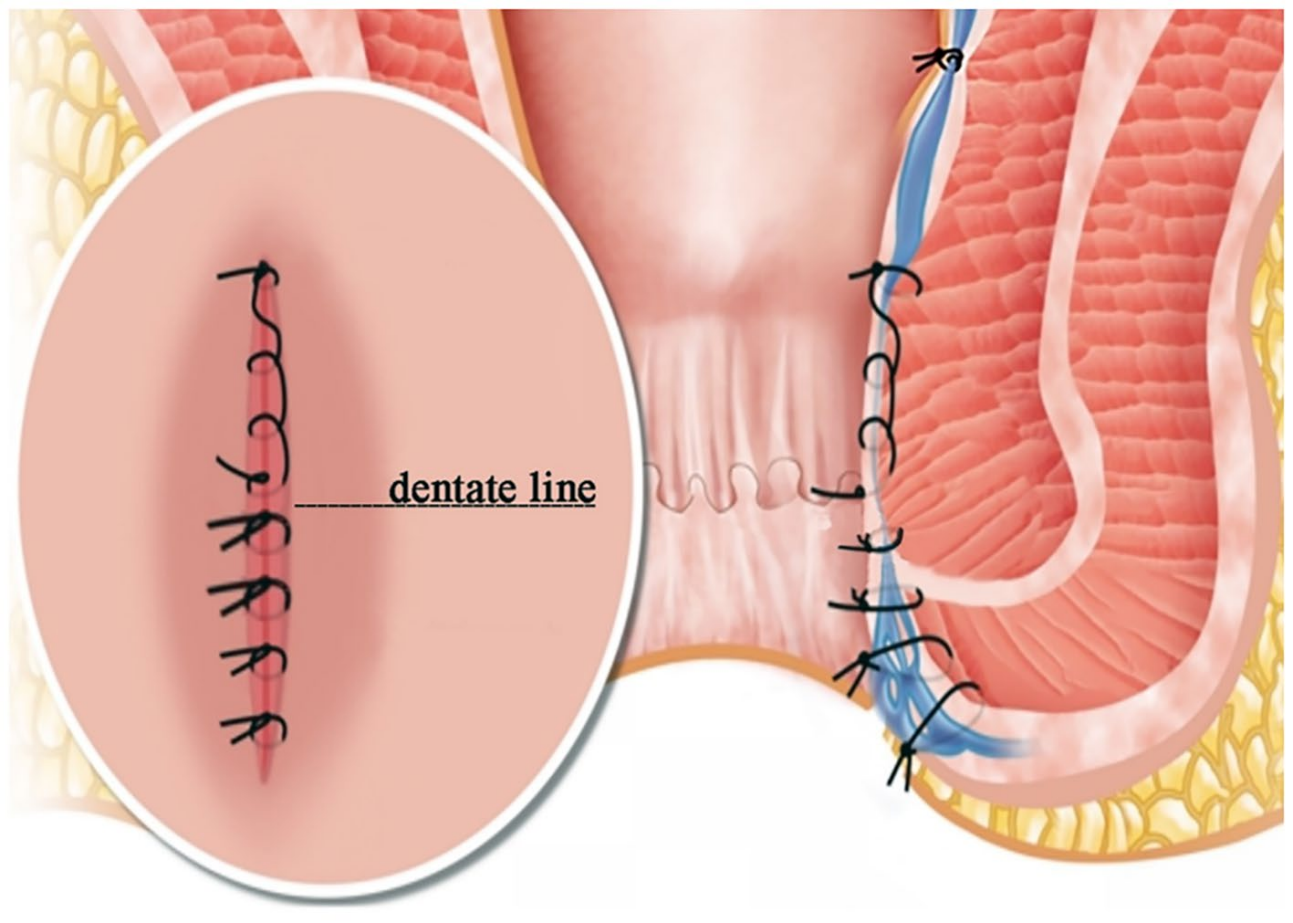
Modified Ferguson hemorrhoidectomy diagram. Note: These images are original and unpublished.

**Figure 3:**
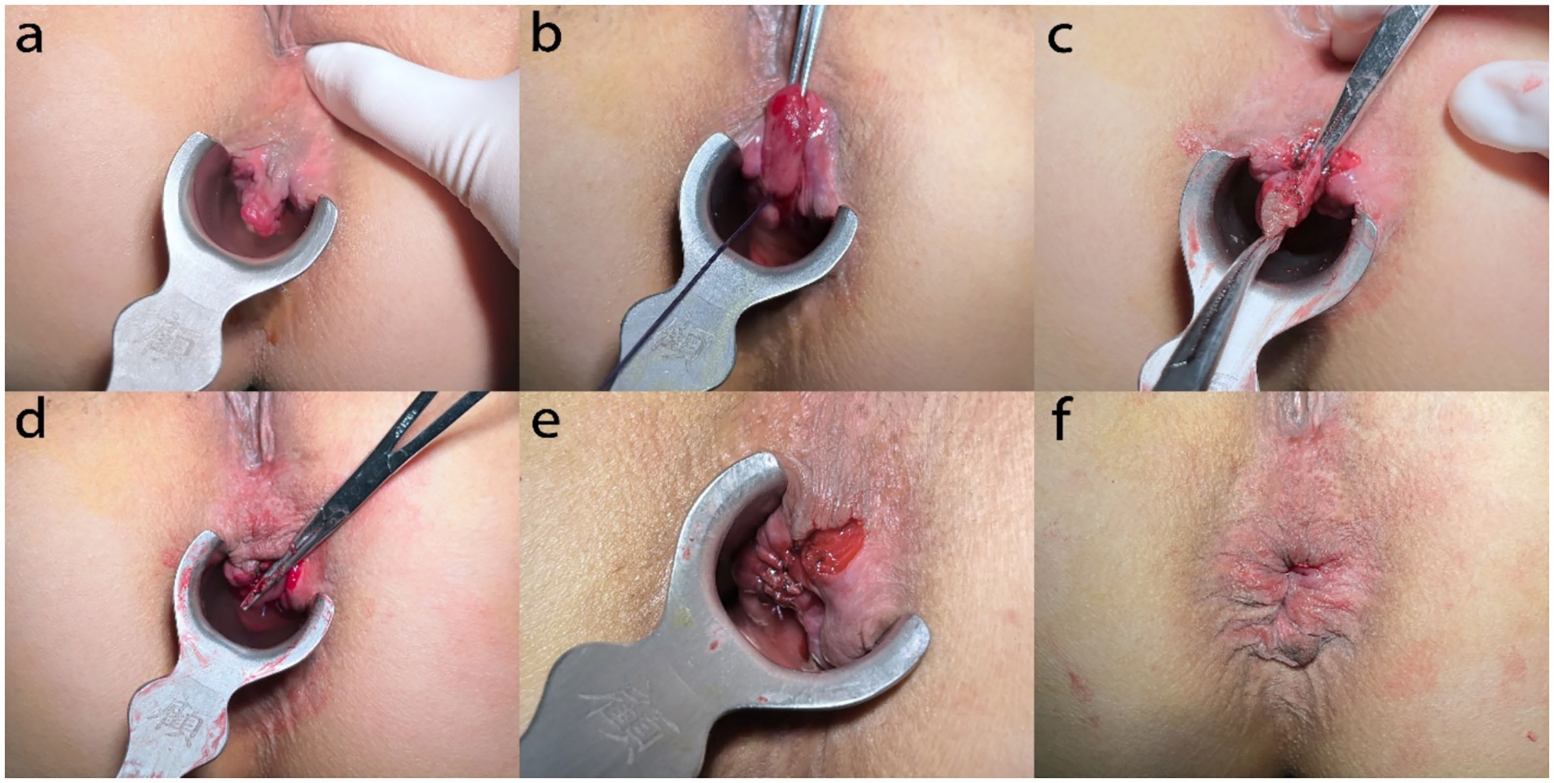
Modified Ferguson procedure. **(A)** Expose the hemorrhoid. **(B)** Ligate the superior hemorrhoidal artery. **(C)** Clamp the hemorrhoid tissue. **(D)** Excise the hemorrhoid tissue along the surface of the internal sphincter. **(E)** Suture continuously from the apex of the internal hemorrhoidectomy wound to the dentate line with 2–0 absorbable suture. **(F)** Intermittent suture under the dentate line with 3–0 fast absorbable suture.

In the MFH group, hemorrhoids were exposed using a Ferguson retractor (33 mm in diameter). Following digital identification, the superior hemorrhoidal artery was ligated with 2–0 absorbable sutures. A V-shaped incision was made at the margin of the external hemorrhoid using surgical scissors. Both external and internal hemorrhoidal tissues were then dissected along the plane of the internal sphincter. The terminal branches of the superior hemorrhoidal artery were identified and ligated based on digital palpation of arterial pulsation at the pedicle, a method validated in prior studies of hemorrhoidal artery ligation. While Doppler-guided identification can enhance precision, digital identification is a well-established, effective, and more widely accessible technique that does not require specialized equipment, aligning with the practical aim of this modification to be broadly applicable. Wound closure was performed in two layers: the proximal segment, from the apex of the wound to the dentate line, was closed continuously using 2–0 polyglactin (Vicryl®) sutures, which provide medium-term wound support. The distal segment, below the dentate line, was approximated with interrupted sutures using 3–0 fast-absorbing polyglactin (Vicryl Rapide®) sutures to minimize suture reaction and tension in the highly sensitive anoderm (Figures 2, 3).

In the MMH group, a V-shaped surgical incision was made depending on the shape of the external hemorrhoid. The skin tissue and varicose veins were dissected until the muscle fiber of the internal sphincter was exposed. The internal hemorrhoid was removed after its pedicle was ligated with 0 silk sutures.

All surgical procedures were performed by one of four attending colorectal surgeons, each with over 5 years of independent practice experience and a personal case volume of more than 100 hemorrhoidectomies prior to the study period. Metronidazole suppository was prescribed postoperatively. Warm water bath on the surgical sites once a day was recommended.

### Primary and secondary outcomes

The primary outcomes were healing time and recurrence rate after a 2-year follow-up period. ‘Healed’ was defined as the point at which the surgical wound was completely epithelialized, with no exudate, and no requirement for further wound care dressing, as confirmed during a proctological examination. Recurrence was defined as either: (1) unchanged or worsened hemorrhoidal disease symptoms compared to baseline, or (2) any subsequent surgical or pharmacological intervention for hemorrhoids during follow-up (15, 16).

Secondary outcomes included:

(1) Hemorrhoidal Disease Symptom Score (HDSS) (17) assessed before and two years after surgery;
(2) Postoperative complications, including pain, acute hemorrhoidal thrombosis, wound infection, hemorrhage requiring hospitalization, urinary retention, anal stenosis, and fecal incontinence;
(3) Operative time and length of hospital stay;
(4) Patient satisfaction and quality of life (QoL) measures.

The HDSS evaluates five symptoms—pain, itching, bleeding, soiling, and swelling/prolapse—with total scores ranging from 0 to 20; higher scores indicate more severe symptoms. HDSS data were collected through outpatient interviews and clinical examinations both preoperatively and during long-term follow-up. Postoperative pain was assessed using a visual analog scale (VAS) (18) on postoperative days 1, 3, and 7, where 0 indicated no pain, 1–3 mild pain, 4–6 moderate pain, and 7–10 severe pain. Fecal incontinence was evaluated with the Wexner Incontinence Score (19). Patient satisfaction was rated on a numerical scale from 0 (“not satisfied”) to 10 (“very satisfied”). Quality of life was measured before and two years after surgery using the Short Health Scale for Hemorrhoidal Disease (SHSHD) (17), which yields a total score between 4 (best QoL) and 28 (worst QoL).

### Sample size and data collection

Sample size calculation was based on the assumption of a recurrence rate of 2% in the MFH group and 8% in the MMH group (20). With an *α* error of 0.05, a *β* error of 0.2, and an assumed loss-to-follow-up rate of 10% in a 2-tailed test, the sample size was 226 patients in each group.

Data on patient characteristics, medical records were obtained from electronic medical charts. The retreatment records, HDSS questionnaire, satisfaction survey and SHS_HD_ score were obtained by clinical follow-up two years after the operation. Two experienced clinical fellows conducted data collection to minimize potential observer bias.

### Statistical analysis

Quantitative data are shown as the mean ± standard deviation, and group comparisons were performed using Student’s t test or the rank-sum test according to the data distribution. Qualitative data are shown as frequency and percentage. The χ^2^ or Fisher’s exact probability tests were used to compare groups of disordered data. The rank-sum test was used to compare groups of graded data. All analyses were conducted with IBM® SPSS® Statistics version 25.0. A two-sided *p*-value of less than 0.05 was considered statistically significant. For outcomes with low event counts, particularly recurrence, standard logistic regression may produce biased estimates. To address this, we employed Firth’s penalized-likelihood method, a bias-reduction technique suitable for small samples, using the logistf package in R (version 4.2.2). The results from this robust sensitivity analysis corroborated the findings from the conventional multivariate model, confirming MFH as an independent protective factor against recurrence.

## Results

### Baseline patient characteristics

A total of 515 patients were enrolled, including 254 treated with MFH and 261 treated with MMH (Figure 1). In the MFH group, 94 patients (37.0%) were male, and 160 (63.0%) were female, with a combined mean age of 41.8 ± 12.0 years. In the MMH group, 100 patients (38.3%) were male, and 161 (61.7%) were female, with a combined mean age of 40.8 ± 12.0 years. The mean follow-up time was 29 months (range 24–36 months). There were no statistically significant differences between the two groups in terms of age, sex, BMI, disease duration, hemorrhoid grade, previous hemorrhoidal surgery or the number of excised hemorrhoids (*p* > 0.05) (Table 1).

**Table 1:** Baseline characteristics of patients.

Index	MFH (*n* = 254)	MMH (*n* = 261)	*p* value
Age, mean (SD), y	41.8 ± 12.0	40.8 ± 12.0	0.33^b^
Gender, *n* (%)			0.76^a^
Female	160 (63.0%)	161 (61.7%)	
Male	94 (37.0%)	100 (38.3%)	
BMI, mean (SD), kg/m^2^	22.7 ± 3.0	22.6 ± 3.2	0.80^b^
Disease duration, *n* (%)			0.37^a^
<3 years	94 (37.0%)	82 (31.4%)	
3-10 years	129 (50.8%)	141 (54.0%)	
>10 years	31 (12.2%)	38 (14.6%)	
Goligher degree, *n* (%)			0.36^a^
II	22 (8.7%)	31 (11.9%)	
III	196 (77.1%)	200 (76.6%)	
IV	36 (14.2%)	30 (11.5%)	
Previous hemorrhoid surgery, *n* (%)	20 (7.9%)	18 (6.9%)	0.67^a^
Hemorrrhoids excised numbers, mean (SD)	1.9 ± 0.7	2.0 ± 0.7	0.19^a^
HDSS before surgery, mean (SD)	7.0 ± 2.5	6.9 ± 2.2	0.92^a^
SHS_HD_ before surgery, mean (SD)	13.8 ± 5.5	13.5 ± 4.7	0.42^*a^

MFH, modified Ferguson hemorrhoidectomy; MMH, Milligan-Morgan hemorrhoidectomy; SD, standard deviation; ^a^χ^2^ test; ^b^Student’s T test.

### Surgical outcomes

After a median follow-up of 29 months, the recurrence rate was significantly lower in the MFH group compared with MMH (1.2% vs. 5.0%, *p* = 0.013). Three MMH patients required reoperation, while further medical treatment for minor symptoms was needed in 3 MFH and 10 MMH cases. HDSS scores decreased significantly in both groups, with lower scores in MFH at two years (0.20 ± 0.50 vs. 0.40 ± 0.90, *p* < 0.001).

MFH achieved faster recovery with a shorter healing time (26.3 ± 4.3 vs. 30.9 ± 3.5 days, p < 0.001), although the operation time was slightly longer (42.6 ± 11.2 vs. 38.7 ± 11.4 min, p < 0.001). Hospitalization duration was similar between groups. Postoperative edema (2.4% vs. 9.6%, *p* = 0.01) and delayed bleeding (0.8% vs. 3.4%, *p* = 0.03) were less frequent in MFH, whereas urinary retention was more common (3.9% vs. 1.1%, *p* = 0.04). No anal stenosis, incontinence, or persistent wound infections occurred in MFH; two MMH patients developed wound infection, with one complicated by perianal abscess.

Patient satisfaction was higher in MFH (9.7 ± 0.7 vs. 9.4 ± 1.2, *p* = 0.002). SHSHD scores improved significantly after surgery in both groups, with sustained benefits during long-term follow-up (Tables 2, 3).

**Table 2:** Postoperative outcomes.

Index	MFH (*n* = 254)	MMH (*n* = 261)	*P* value
Healing time	26.3 ± 4.3	30.9 ± 3.5	0.001^*a^
Overall recurrence, *n* (%)	3(1.2%)	13(5.0%)	0.013^*a^
Need of redo surgery	0	3(1.2%)	
Mild symptoms require medication	3(1.2%)	10(3.8%)	0.055 ^a^
HDSS 2 year after surgery, mean (SD)	0.2 ± 0.5	0.4 ± 0.9	0.001^*a^
Postoperative pain VAS, mean (SD)			
1 day after surgery	5.0 ± 1.2	4.8 ± 1.3	0.21^b^
3 day after surgery	1.2 ± 2.2	0.9 ± 1.8	0.07^b^
7 day after surgery	0.7 ± 1.8	0.7 ± 1.8	0.86^b^
Operation time, mean (SD), min	42.6 ± 11.2	38.7 ± 11.4	0.001^*a^
Hospitalization time, mean (SD), day	7.6 ± 2.6	8.0 ± 2.6	0.06^b^
Satisfaction, mean (SD)	9.7 ± 0.7	9.4 ± 1.2	0.002^*b^
SHS_HD_ 2 year after surgery, mean (SD)	4.8 ± 1.2	5.0 ± 1.9	0.25^a^

MFH, modified Ferguson hemorrhoidectomy; MMH, Milligan-Morgan hemorrhoidectomy; SD, standard deviation; ^a^χ^2^ test; ^b^Student’s T test; ^*^P is significant.

**Table 3:** Postoperative complication profiles with between-group comparisons.

Complication	MFH (*n* = 254)	MMH (*n* = 261)	Risk difference (MFH - MMH), % (95% CI)	*P* value
Edema	6 (2.4%)	25 (9.6%)	−7.2 (−11.0 to −3.4)	0.01
Delayed bleeding	2 (0.8%)	9 (3.4%)	−2.7 (−5.0 to −0.3)	0.03
Urinary retention	10 (3.9%)	3 (1.1%)	2.8 (0.1 to 5.5)	0.04
Wound infection	0 (0.0%)	2 (0.8%)	−0.8 (−2.3 to 0.7)	0.49
Anal stenosis	0 (0.0%)	0 (0.0%)	-	-
Fecal incontinence	0 (0.0%)	0 (0.0%)	-	-

### Kaplan–Meier analysis

**Figure 4:**
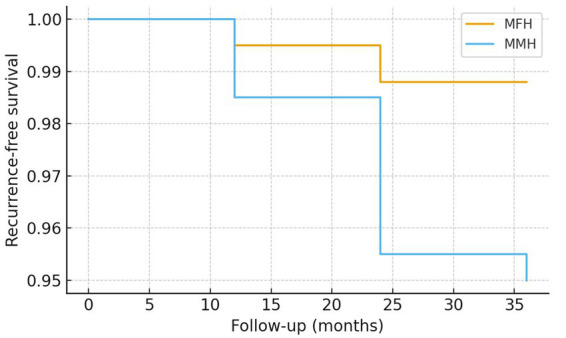
Kaplan–Meier curves of recurrence-free survival after hemorrhoidectomy. Kaplan–Meier analysis demonstrated a consistently higher recurrence-free survival rate in the modified Ferguson hemorrhoidectomy (MFH) group compared to the Milligan–Morgan hemorrhoidectomy (MMH) group during the 36-month follow-up period (98.8% vs. 95.0% at 36 months).

Kaplan–Meier analysis showed that the recurrence-free survival rate was consistently higher in the MFH group compared to the MMH group throughout the follow-up period (36 months: 98.8% vs. 95.0%) (Figure 4).

### Subgroup analyses and complication profiles

Subgroup analyses showed similar trends across baseline characteristics. Recurrence remained lower in MFH for both males (1.1% vs. 4.9%) and females (1.3% vs. 5.0%), in younger (<40 years, 1.0% vs. 4.8%) and older patients (≥40 years, 1.3% vs. 5.2%), and in both Goligher grade II–III (0.9% vs. 4.6%) and grade IV patients (2.8% vs. 6.7%). Notably, no recurrences were observed in MFH patients with a history of prior hemorrhoid surgery, compared with 5.6% in the MMH group.

Complication profiles were distinct between groups. Edema (2.4% vs. 9.6%, *p* = 0.01) and delayed bleeding (0.8% vs. 3.4%, *p* = 0.03) occurred less frequently in MFH, whereas urinary retention was more common (3.9% vs. 1.1%, *p* = 0.04). No patients in either group experienced anal stenosis or fecal incontinence (Tables 4, 5).

**Table 4:** Subgroup analysis of recurrence after hemorrhoidectomy.

Subgroup	MFH (n/N, %)	MMH (*n*/*N*, %)	*P* value
Male	1/94 (1.1%)	5/100 (4.9%)	0.21
Female	2/160 (1.3%)	8/161 (5.0%)	0.12
Age <40 years	1/100 (1.0%)	4/82 (4.8%)	0.18
Age ≥40 years	2/154 (1.3%)	9/179 (5.2%)	0.11
Goligher II–III	2/218 (0.9%)	9/231 (4.6%)	0.05
Goligher IV	1/36 (2.8%)	2/30 (6.7%)	0.45
Prior surgery	0/20 (0%)	1/18 (5.6%)	0.29

**Table 5:** Postoperative complications.

Complication	MFH (*n*/*N*, %)	MMH (*n*/*N*, %)	*P* value
Edema	6/254 (2.4%)	25/261 (9.6%)	0.01
Delayed bleeding	2/254 (0.8%)	9/261 (3.4%)	0.03
Urinary retention	10/254 (3.9%)	3/261 (1.1%)	0.04

### Effect sizes and multivariate analysis

MFH significantly reduced recurrence risk (OR 0.24, 95% CI 0.07–0.84, *p* = 0.013), shortened healing time by 4.6 days (95% CI 3.8–5.4, *p* = 0.001), and lowered the risk of postoperative edema (OR 0.23, 95% CI 0.09–0.56, p = 0.01). Multivariate logistic regression confirmed MFH as an independent protective factor against recurrence (adjusted OR 0.26, 95% CI 0.08–0.91, *p* = 0.035) (Table 6).

**Table 6:** Effect sizes and regression results.

Outcome	Effect size	95% CI	*P* value
Recurrence	OR 0.24	0.07–0.84	0.013
Healing time (days)	Mean difference −4.6	3.8–5.4	0.001
Edema complication	OR 0.23	0.09–0.56	0.01
Multivariate regression (recurrence)	Adjusted OR 0.26	0.08–0.91	0.035

### Long-term follow-up

Follow-up rate: Of 515 patients, 492 (95.5%) completed the full follow-up, while 23 patients (4.5%) were lost. Quality of life: SHSHD scores improved significantly and remained stable over time. At baseline, mean SHSHD was 13.8 ± 5.5 in MFH and 13.5 ± 4.7 in MMH. After 2 years, scores decreased to 4.8 ± 1.2 and 5.0 ± 1.9, respectively, with no significant group difference (*p* = 0.25).

Sustained improvement: Line chart analyses of SHSHD and HDSS scores demonstrated that symptom relief was maintained throughout the 24–36 month follow-up, with no evidence of late deterioration.

## Discussion

Although numerous minimally invasive techniques have been developed, Ferguson hemorrhoidectomy remains one of the most effective treatments for HD (21). Our study validated its low recurrence rate and high patient satisfaction outcome. Moreover, we reduced the postoperative complications by modifying the surgical procedure.

Studies of transanal hemorrhoidal dearterialization (THD) have revealed favorable results (22–24). Artery ligation can reduce the arterial inflow to the hemorrhoids while keeping the venous outflow intact, the tension within the anal cushions drops, and pile collapse, bleeding and pain cease (23). Hemorrhoidal artery ligation can be effectively completed under digital guidance without the need for Doppler signal, as demonstrated in several studies that report comparable efficacy and safety between digital and Doppler-assisted ligation (24, 25). The digital method relies on palpation of arterial pulsations at the pedicle, which is both feasible and reliable in most patients, while avoiding the cost and logistical requirements of Doppler equipment. This approach aligns with the goal of making the modified technique accessible in diverse clinical settings. Ligation before hemorrhoidectomy reduces intraoperative and postoperative bleeding. Postoperative bleeding is a serious surgical complication that occurs 4–12 days after surgery at a rate of 0.9% ~ 5.2% (26, 27), while the incidence in our study was 0.8% (2 cases) with MFH.

Closed hemorrhoidectomy resulted in less pain and a shorter healing time than open hemorrhoidectomy (28). In traditional Ferguson hemorrhoidectomy, continuous sutures are used to close the surgical wound, which could be accompanied by the risk of wound dehiscence and infection (7, 29). Therefore, we used a 3–0 fast absorption line of intermittent sutures for the wound surface below the dentate line. The suture method was changed to relieve tension. Fine and rapidly absorbed sutures reduced the suture reaction. No dehiscence or wound infection were observed in the MFH group; a higher incidence of both of these complications was reported (5% ~ 11.8%) in previous studies (9, 30).

Traditional three-quadrant excision of HD was not necessary (31). This concept is supported by recent studies advocating for tailored, limited excisions. For example, Tomasicchio et al. (32) demonstrated in an outpatient setting that a tailored hemorrhoidectomy approach—removing only symptomatic hemorrhoidal piles—is safe and effective, with low recurrence rates and high patient satisfaction, further reinforcing the rationale for our limited excision strategy. Hayssen et al. found that limited hemorrhoidectomy treating only problematic hemorrhoids does not result in a significantly higher incidence of recurrent anorectal symptoms or the need for additional treatment for the remaining quadrants (33). We performed limited hemorrhoidectomy with a recurrence rate of only 1.5% in the MFH group. Jeong HY et al. reported that having more than four hemorrhoid piles was an independent risk factor for postoperative delayed bleeding (34). The incidence of postoperative delayed bleeding here was only 0.8% (2 cases) in MFH and 1.1% (3 cases) in MMH. However, no more than three piles were typically excised, potentially explaining this low incidence.

Urinary retention is a common complication following hemorrhoidectomy (35). In this study, the incidence of postoperative urinary retention was higher in the MFH group than in the MMH group. According to Bailey and Ferguson, restricting perioperative intravenous or oral fluid intake can reduce the risk of this complication (36). As our protocol did not impose such fluid restrictions, and the MFH procedure was associated with a longer operative time—leading to greater postoperative fluid supplementation—this likely explains the higher incidence observed in the MFH group. The observed dissociation between postoperative edema and urinary retention—wherein the MFH group had a lower incidence of edema but a higher incidence of urinary retention—suggests that factors other than local anal swelling may have contributed to urinary retention. As noted earlier, the longer operative time in the MFH group likely led to greater perioperative fluid administration, which is a well-established independent risk factor for postoperative urinary retention. Thus, while edema may contribute to urinary retention via local compression or reflex mechanisms, the predominant driver in our cohort appears to be related to fluid management and surgical duration rather than the degree of perianal edema.

The MFH hemorrhoidectomy procedure maintains the advantages of low recurrence. Meanwhile, patients in the MFH group had less postoperative pain and margin edema than those in the MMH group. Thus, the modified method achieved satisfactory results. We hypothesize that the outcomes might be related to the use of intermittent sutures on the wound under the dentate line, which reduces anal tension. However, further clinical studies are needed to confirm this hypothesis.

It is worth noting that regarding the relationship between postoperative edema and pain, while these two factors are often correlated, the dissociation observed in our study—where the MMH group had significantly more edema but similar pain scores to the MFH group—may be explained by the technical modifications of the MFH procedure. Specifically, the use of interrupted sutures with fine, rapidly-absorbable material below the dentate line in the MFH technique, as opposed to the open wounds in MMH, might have introduced a different source of discomfort. It is plausible that the sutures in the highly sensitive anoderm of MFH patients created a baseline level of tension-related or suture-material-related pain that, while well-managed, offset the pain-reducing benefit of having less edema. Consequently, the overall reported pain scores between the two groups became comparable. This interpretation suggests that the MFH technique may have exchanged the pain of significant edema and tissue inflammation (more common in MMH) for a milder, suture-related discomfort, ultimately leading to a similar pain experience but through different mechanistic pathways. This hypothesis, which aligns with the finding of higher patient satisfaction in the MFH group despite similar pain scores, warrants further investigation.

This study has a larger sample size than similar studies. Meanwhile, it has some limitations. First, this is a single-center retrospective analysis constrained by such studies’ inherent limitations. Most notably, the choice between MFH and MMH was based on surgeon preference rather than randomization, which introduces a potential for selection bias and unmeasured confounding. Although our multivariate analysis adjusted for available baseline characteristics and found no significant differences between groups, residual confounding may persist. Second, the procedures were performed by four surgeons, and while all were highly experienced, differences in individual technique or skill could represent an unaccounted ‘surgeon effect’. We did not perform a formal sensitivity analysis to account for the operator as a cluster variable, and this should be considered when interpreting the results. Third, methodologically while our modified technique focused on reducing tension below the dentate line, the sutures in the proximal segment could indeed contribute to postoperative discomfort. In light of this, we will incorporate a refinement to the MFH technique in our future practice and studies: namely, to minimize the number of sutures or utilize even finer-gauge absorbable sutures in the region above the dentate line, with the specific aim of further reducing suture-induced pain. Finally, the two-year follow-up period, while a minimum, was relatively short for assessing very long-term recurrence. Prospective studies with a randomized design, longer follow-up time, and protocolized surgeon assignment remain necessary to further validate the effect of the modified surgery.

## Conclusion

This study demonstrates that MFH is superior to the MMH for treating grade II–IV hemorrhoids, offering a significantly lower recurrence rate, shorter healing time, reduced risks of postoperative edema and bleeding, and higher patient satisfaction. Although this was a retrospective single-center study with potential for selection bias, the results strongly indicate that MFH is a safe, effective, and valuable refinement of conventional hemorrhoidectomy. The technical modifications of arterial ligation and segmented suturing likely contribute to its improved outcomes. We recommend MFH as a standard surgical option, pending further prospective validation. Furthermore, future iterations of this technique will focus on optimizing the suturing strategy above the dentate line to achieve further reductions in postoperative pain.

## Data Availability

The raw data supporting the conclusions of this article will be made available by the authors, without undue reservation.
